# Stigmatizing Attitudes Across Cybersuicides and Offline Suicides: Content Analysis of Sina Weibo

**DOI:** 10.2196/36489

**Published:** 2022-04-08

**Authors:** Ang Li, Dongdong Jiao, Tingshao Zhu

**Affiliations:** 1 Department of Psychology Beijing Forestry University Beijing China; 2 Key Laboratory of Behavioral Science Institute of Psychology Chinese Academy of Sciences Beijing China; 3 National Computer System Engineering Research Institute of China Beijing China; 4 Department of Psychology University of Chinese Academy of Sciences Beijing China

**Keywords:** stigma, cybersuicide, livestreamed suicide, linguistic analysis, social media

## Abstract

**Background:**

The new reality of cybersuicide raises challenges to ideologies about the traditional form of suicide that does not involve the internet (offline suicide), which may lead to changes in audience’s attitudes. However, knowledge on whether stigmatizing attitudes differ between cybersuicides and offline suicides remains limited.

**Objective:**

This study aims to consider livestreamed suicide as a typical representative of cybersuicide and use social media data (Sina Weibo) to investigate the differences in stigmatizing attitudes across cybersuicides and offline suicides in terms of attitude types and linguistic characteristics.

**Methods:**

A total of 4393 cybersuicide-related and 2843 offline suicide-related Weibo posts were collected and analyzed. First, human coders were recruited and trained to perform a content analysis on the collected posts to determine whether each of them reflected stigma. Second, a text analysis tool was used to automatically extract a number of psycholinguistic features from each post. Subsequently, based on the selected features, a series of classification models were constructed for different purposes: differentiating the general stigma of cybersuicide from that of offline suicide and differentiating the negative stereotypes of cybersuicide from that of offline suicide.

**Results:**

In terms of attitude types, cybersuicide was observed to carry more stigma than offline suicide (*χ^2^*_1_=179.8; *P*<.001). Between cybersuicides and offline suicides, there were significant differences in the proportion of posts associated with five different negative stereotypes, including *stupid and shallow* (*χ^2^*_1_=28.9; *P*<.001), *false representation* (*χ^2^*_1_=144.4; *P*<.001), *weak and pathetic* (*χ^2^*_1_=20.4; *P*<.001), *glorified and normalized* (*χ^2^*_1_=177.6; *P*<.001), and *immoral* (*χ^2^*_1_=11.8; *P*=.001). Similar results were also found for different genders and regions. In terms of linguistic characteristics, the *F*-measure values of the classification models ranged from 0.81 to 0.85.

**Conclusions:**

The way people perceive cybersuicide differs from how they perceive offline suicide. The results of this study have implications for reducing the stigma against suicide.

## Introduction

### Background

Suicide remains one of the leading causes of death worldwide according to the latest estimates released by the World Health Organization [1]. Mental illness stigma refers to the negative stereotyping of people with mental illnesses that may lead to discrimination [2]. Although there is often a stigma associated with all mental illnesses, suicide can be especially stigmatized, dismissed as “merely attention-seeking gesturers” [3]. Stigma against suicide can lead to a reduced likelihood of receiving treatment and an increased risk of death by suicide [4,5]. Therefore, lowering stigma can improve mental health outcomes. Stigma reduction efforts are more likely to be effective when targeting specific mental health problems [6-8]. Therefore, it is crucial to understand the negative stereotypes people often associate with suicide and then design antistigma campaigns accordingly.

The internet facilitates self-disclosure and social connection, giving rise to an emerging form of suicide (ie, cybersuicide). Unlike the traditional form of suicide that does not involve the internet (ie, offline suicide), cybersuicide covers a broad range of internet-mediated suicidal behaviors and phenomena, including livestreamed suicide [9,10]. The internet increases people’s willingness to disclose more about themselves and offers highly interactive platforms for interpersonal communication (eg, Twitter and Sina Weibo). Therefore, on the internet, people with suicidal intentions are motivated to share and communicate their thoughts on suicide with others and are able to maintain active contact with their social network in the last moments of life, although members of social networks do not live close to them. In livestreamed suicide, for example, through various media (text messages, pictures, videos, and voice notes), the internet enables people with suicidal intentions to broadcast their suicides to their entire social network before death and allows real time interaction between people with suicidal intentions and their audience. This means that cybersuicide makes a very personal and private act highly public and greatly enhances the interaction between people with suicidal intentions and their audience, which raises challenges to long-held ideas and beliefs about offline suicide [11-13] and facilitates the creation of emerging cultures around suicide [9,10,14,15]. It is well known that social and cultural factors can influence attitudes toward mental health problems, including suicide [16-18]. Therefore, it is not surprising that cybersuicide, which has radically transformed the sociocultural context of suicide, may lead to changes in audience’s attitudes, suggesting the need for further examination of the differences in stigmatizing attitudes across cybersuicides and offline suicides.

The livestreamed suicide is commonly considered as one of the most notable and representative types of cybersuicide, particularly in China [9,19]. From 2003 to 2016, at least 193 livestreamed suicide incidents occurred in China [20]. Therefore, considerable research efforts have been directed toward understanding the specific types of attitudes associated with livestreamed suicide. As social media allow users to freely disclose their feelings and thoughts and include vast quantities of publicly available data, many studies used human coders to analyze relevant social media data (eg, posts with relevant keywords and audience-generated messages related to relevant suicide incidents) and concluded that the public may react strongly against livestreamed suicide [21-24]. Besides, a study found a distinctive type of negative stereotype associated with livestreamed suicide (ie, false representation stigma, a misleading belief that people livestreaming their suicides do not really want to kill themselves) [25], which was not mentioned in previous studies regarding offline suicide. The results of these studies imply that the public may react differently to cybersuicides and offline suicides. However, to our knowledge, no research has been conducted to directly investigate the differences in stigmatizing attitudes toward cybersuicides and offline suicides. Furthermore, attitudes can manifest not only in specific attitude types (what it says exactly) but also in linguistic characteristics of language expressions (how it is said) [26-29]. Recent studies confirmed that health-related stigmatizing attitudes (eg, Alzheimer disease, depression, and livestreamed suicide) can be identified by analyzing linguistic characteristics of expressions in social media posts [30-32]. More importantly, the differences in stigmatizing attitudes across mental health problems can be reflected in different patterns of language use as well [33]. Therefore, apart from the differences in types of stigma, the differences in the linguistic characteristics of stigmatizing expressions also need to be investigated.

### Objective

To address these concerns by analyzing social media data (Sina Weibo, a Chinese social media site that is similar to Twitter), this study attempts to directly and systematically investigate the differences in stigmatizing attitudes across cybersuicides and offline suicides in terms of attitude types and linguistic characteristics, respectively.

It is worth noting that cybersuicide is a new and developing form of suicide. The public may not be equally familiar with different types of cybersuicide. In China, because of the prevalence and media coverage of livestreamed suicide, compared with other types of cybersuicide, the public is expected to be more familiar with and more likely to discuss livestreamed suicide on social media. Therefore, to collect sufficient social media data for further analysis, this study aims to consider livestreamed suicide as a typical representative of cybersuicide and compare it with offline suicide.

## Methods

### Research Process

**Figure 1:**
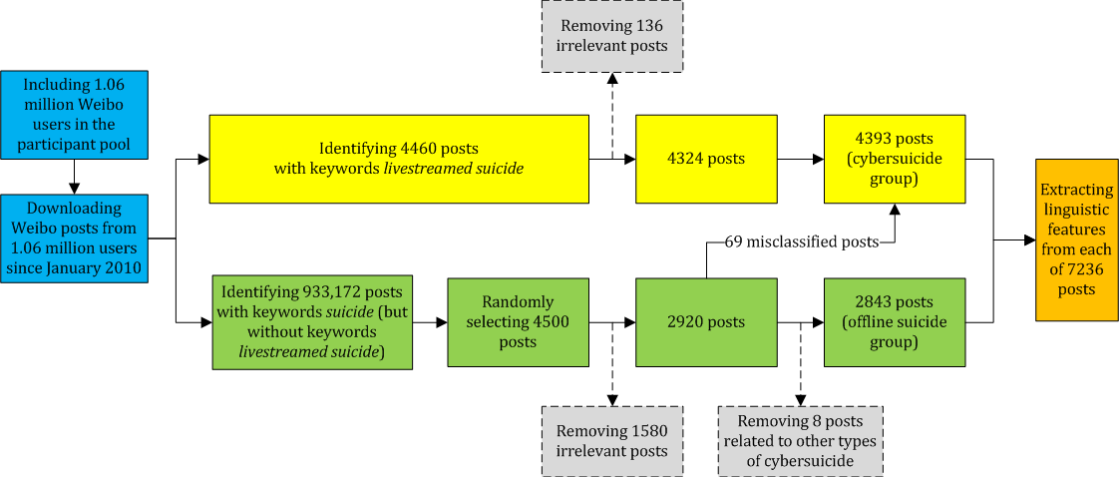
Procedures of data collection and preprocessing.

The research process included the following three steps: (1) data collection, (2) data preprocessing, and (3) data analysis. The data collection and preprocessing procedures are shown in Figure 1.

#### Data Collection

First, a participant pool of active Weibo users was created. According to a previous study, 1,953,485 active Weibo users were identified as potential participants [34]. The application programming interface (API) platform of Sina Weibo enables programmatic access to Weibo data that users choose to share with the public. As Sina Weibo places limits and quotas on API requests, among all potential participants, 1.06 million active users with available data were finally included in the participant pool.

Second, a database of Weibo posts was constructed. In May 2020, using API, a vast amount of publicly available Weibo posts from 1.06 million active users in the participant pool since their registration (the 2020 official numbers of monthly and daily active users: 511 million and 224 million, respectively [35]), were downloaded.

Third, several relevant Weibo posts were identified through database searches. To obtain the posts reflecting attitudes toward cybersuicides and offline suicides, two sets of keywords were used to search the database, including *livestreamed suicide* (*直播自杀* and *自杀直播*) and *suicide* (*自杀*). It is worth noting that although cybersuicide is a rapidly developing form of suicide, an overwhelming majority of suicide incidents still occur offline. For example, between 2003 and 2016, 193 incidents of livestreamed suicide occurred in China [20], whereas in 2019, the number of suicides in China had reached 116,324 [1]. Therefore, unless otherwise stated, the Chinese people commonly use the term *suicide* to refer to traditional offline suicide.

The process of database searches included the following three steps: (1) a total of 4460 posts with keywords *livestreamed suicide* were searched and obtained (cybersuicide group); (2) a total of 933,172 posts with keywords *suicide* (but without keywords *livestreamed suicide*) were searched and obtained (offline suicide group); (3) to balance the number of posts in each group, using simple random sampling, 4500 posts were randomly selected from 933,172 posts in the offline suicide group (cybersuicide: 4460 posts and offline suicide: 4500 posts).

#### Data Preprocessing

After data collection, preprocessing was performed on the raw data to prepare them for further analysis.

First, to exclude irrelevant posts and reclassify misclassified posts, manual scrutiny of the collected data was conducted.

In this study, irrelevant posts were considered as (1) posts that depicted suicides in fictional works (eg, movies, television programs, and novels), (2) posts that focused on suicides in nonhuman animals (eg, dogs), and (3) posts that used suicide-related keywords for nonsuicidal purposes (eg, making a bet). After the removal of irrelevant posts, 7244 posts (cybersuicide: 4460 – 136 = 4324 and offline suicide: 4500 − 1580 = 2920) remained.

Subsequently, 77 posts in the offline suicide group were reclassified as posts related to cybersuicide (livestreamed suicide: n=69, 90% posts; suicide *game*: n=4, 5% posts; prosuicide website and forum: n=2, 3% posts; and internet suicide pact: n=2, 3% posts). As this study primarily focused on livestreamed suicide rather than other types of cybersuicide, 8 posts related to the other 3 types of cybersuicide were excluded from further analysis.

Therefore, the final sample of this study included 7236 posts (cybersuicide: 4324 + 69 = 4393; offline suicide: 2920 – 69 – 8 = 2843). The demographic characteristics of the participants in the final sample are presented in Table 1.

**Table 1:** Demographics of participants.

Demographics		All Weibo posts (N=7236), n (%)	Cybersuicide (n=4393), n (%)	Offline suicide (n=2843), n (%)
**Gender**				
	Male	4062 (56.14)	2473 (56.29)	1589 (55.89)
	Female	3174 (43.86)	1920 (43.71)	1254 (44.11)
**Regions**				
	North China	1312 (18.13)	812 (18.48)	500 (17.59)
	Northeast China	299 (4.13)	191 (4.35)	108 (3.8)
	East China	2277 (31.47)	1330 (30.28)	947 (33.31)
	Central China	374 (5.17)	233 (5.3)	141 (4.96)
	South China	1345 (18.59)	801 (18.23)	544 (19.13)
	Southwest China	493 (6.81)	330 (7.51)	163 (5.73)
	Northwest China	224 (3.1)	133 (3.03)	91 (3.2)
	International and unspecified	912 (12.6)	563 (12.82)	349 (12.28)

Second, to extract psycholinguistic features from each of the 7236 posts automatically, the Simplified Chinese version of Linguistic Inquiry and Word Count software was used. The Simplified Chinese version of Linguistic Inquiry and Word Count is a reliable and valid text analysis tool for the automatic estimation of word frequency in multiple psychologically meaningful categories, including linguistic processes (eg, personal pronouns), psychological processes (eg, affective processes), personal concerns (eg, achievement), spoken categories (eg, assent), and punctuation categories (eg, periods) [36]. After feature extraction, the standardized values of psycholinguistic features were estimated for further analysis.

#### Data Analysis

##### Human Coding

To explore the differences in types of stigmatizing attitudes across cybersuicides and offline suicides, a content analysis was performed on all 7236 posts to determine whether each of them reflected stigma. The coding framework was developed based on expert consensus and available evidence. Specifically, in this study, a researcher (AL) reviewed relevant studies [25,37] and then performed an inductive content analysis on all 7236 posts to develop an initial coding framework. Subsequently, another two researchers (DJ and TZ) provided feedback to emend the initial framework. Using the amended framework (Table S1 in Multimedia Appendix 1), 2 independent human coders were recruited and trained to analyze all 7236 posts. The levels of agreement between the 2 coders were measured by Cohen *k* coefficient, and disagreements were resolved by the decisions of a researcher (AL). All individuals who participated in developing the coding framework and performing manual coding of posts had considerable experience in coding qualitative materials.

##### Construction of Classification Models

To explore the linguistic differences in stigmatizing expressions across cybersuicides and offline suicides, 2 groups of classification models were built using Waikato Environment for Knowledge Analysis (version 3.9.4; University of Waikato) software. Waikato Environment for Knowledge Analysis provides tools for developing machine learning techniques and applying them to practical data mining problems.

The first group of classification models was built to investigate whether linguistic differences existed in the expression of stigma in general (ie, cybersuicide-related or offline suicide-related stigma as a whole) between cybersuicides and offline suicides. The human coding results were considered as the ground truth for the validation of the classification models. In this study, an imbalanced data problem existed. For example, the number of stigmatizing posts belonging to the offline suicide class (minority class: 588 posts) was obviously lower than those belonging to the cybersuicide class (majority class: 1556 posts). Imbalanced data sets pose a challenge for machine learning modeling, as this problem may result in models with poor predictive performance, especially for the minority class. To handle this problem, using simple random sampling, a certain number of posts were randomly selected from the majority class to obtain a well-balanced data set. Subsequently, to improve classification accuracy, a subset of psycholinguistic features was selected for use in model construction. Specifically, a series of 2-tailed independent *t* tests were conducted to compare the values of all extracted psycholinguistic features between stigmatizing posts in the cybersuicide and offline suicide groups, and then effect sizes (Cohen *d* coefficient) were computed from the estimated *t* values. Features that were statistically significant at .05 and had a Cohen *d* >0.20 or <−0.20 were considered as key features. Finally, using four different machine learning algorithms (Naïve Bayes, support vector machine, multilayer perceptron neural network, and random forest [RF]), 4 classification models were constructed based on the selected key features. Using a 5-fold cross-validation technique, the classification performance of the established models was evaluated in terms of precision, recall, and *F*-measure.

It is worth noting that the good classification performance of models in the first group may be attributed to the existence of differences in the amount and distribution of negative stereotypes across cybersuicides and offline suicides rather than the existence of linguistic differences in stigmatizing expressions across cybersuicides and offline suicides. To clarify this issue, the second group of classification models was built to investigate whether linguistic differences existed in the expression of certain negative stereotypes across cybersuicides and offline suicides. To obtain sufficient data for further analysis, in this study, posts reflecting two negative stereotypes (ie, *stupid and shallow* and *glorified and normalized*) were examined. Well-balanced data sets, key features, and classification models were obtained using the aforementioned methods.

### Ethics Approval

The study protocol was reviewed and approved by the institutional review board of the Institute of Psychology, Chinese Academy of Sciences (protocol number: H15009). Informed consent was not obtained, as this study was based on publicly available data and involved no personally identifiable data collection or analysis.

## Results

### Human Coding

The Cohen *k* coefficients for *attitudes* and *negative stereotypes* reached 0.88 and 0.81, respectively, indicating almost perfect agreement [38]. The results of human coding are presented in Table 2.

**Table 2:** Results of human coding (N=7236).

Categories			Cybersuicide, n (%)		Offline suicide, n (%)
**Attitudes**			4393 (100)		2843 (100)
	Stigmatizing	1556 (35.4)		588 (20.7)	
	Nonstigmatizing	2837 (64.6)		2255 (79.3)	
**Negative stereotypes**			1556 (100)		588 (100)
	Weak and pathetic	114 (7.3)		80 (13.6)	
	Self-centered	97 (6.2)		44 (7.5)	
	Stupid and shallow	528 (33.9)		129 (21.9)	
	False representation	387 (24.9)		13 (2.2)	
	Glorified and normalized	148 (9.5)		195 (33.2)	
	Immoral	111 (7.1)		69 (11.7)	
	Strange	59 (3.8)		14 (2.4)	
	Embarrassing	24 (1.5)		7 (1.2)	
	Vengeful	40 (2.6)		9 (1.5)	
	Mad	48 (3.1)		28 (4.8)	

For stigma in general, posts on cybersuicide were more likely than posts on offline suicide to contain stigmatizing expressions (*χ^2^*_1_=179.8; *P*<.001). Similar results were found for different genders and regions, including men (*χ^2^*_1_=66.7; *P*<.001), women (*χ^2^*_1_=121.0; *P*<.001), North China (NC; *χ^2^*_1_=37.2; *P*<.001), East China (EC; *χ^2^*_1_=56.4; *P*<.001), Central China (CC; *χ^2^*_1_=10.4; *P*=.001), South China (SC; *χ^2^*_1_=37.6; *P*<.001), and Southwest China (SWC; *χ^2^*_1_=11.8; *P*=.001).

For negative stereotypes, posts on cybersuicide were often coded as *stupid and shallow* (528/1556, 33.93%) and *false representation* (387/1556, 24.87%), whereas posts on offline suicide were often coded as *glorified and normalized* (195/588, 33.2%) and *stupid and shallow* (129/588, 21.9%). Furthermore, posts on cybersuicide were more likely than posts on offline suicide to be coded as *stupid and shallow* (*χ^2^*_1_=28.9; *P*<.001) and *false representation* (*χ^2^*_1_=144.4; *P*<.001), whereas posts on offline suicide were more likely than posts on cybersuicide to be coded as *weak and pathetic* (*χ^2^*_1_=20.4; *P*<.001), *glorified and normalized* (*χ^2^*_1_=177.6; *P*<.001), and *immoral* (*χ^2^*_1_=11.8; *P*=.001). Similar results were found for different genders and regions. Specifically, significant differences in the proportions of posts coded as *weak and pathetic* were observed for posts by women (*χ^2^*_1_=21.0; *P*<.001) and from NC (*χ^2^*_1_=10.6; *P*=.001) and EC (*χ^2^*_1_=3.9; *P*=.048). Significant differences in the proportions of posts coded as *stupid and shallow* were observed for posts by men (*χ^2^*_1_=12.6; *P*<.001) and women (*χ*^2^_1_=16.0; *P*<.001) and from NC (*χ^2^*_1_=9.1; *P*=.003), EC (*χ^2^*_1_=7.9; *P*=.005), SC (*χ^2^*_1_=5.4; *P*=.02), and SWC (*χ^2^*_1_=8.0; *P*=.005). Significant differences in the proportions of posts coded as *false representation* were observed for posts by men (*χ^2^*_1_=86.4; *P*<.001) and women (*χ^2^*_1_=59.5; *P*<.001) and from NC (*χ^2^*_1_=25.5; *P*<.001), Northeast China (Fisher exact test: *P*=.004), EC (*χ^2^*_1_=48.2; *P*<.001), CC (Fisher exact test: *P*=.006), SC (*χ^2^*_1_=16.5; *P*<.001), SWC (*χ^2^_1_*=8.1; *P*=.004), and Northwest China (Fisher exact test: *P*=.003). Significant differences in the proportions of posts coded as *glorification and normalized* were observed for posts by men (*χ^2^*_1_=127.3; *P*<.001) and women (*χ^2^*_1_=55.1; *P*<.001) and from NC (*χ^2^*_1_=37.5; *P*<.001), Northeast China (Fisher exact test: *P*<.001), EC (*χ^2^*_1_=60.7; *P*<.001), CC (Fisher exact test: *P*=.01), SC (*χ^2^*_1_=17.6; *P*<.001), and SWC (*χ^2^*_1_=21.5; *P*<.001). Significant differences in the proportions of posts coded as *immoral* were observed for posts by men (*χ^2^*_1_=7.8; *P*=.005) and women (*χ^2^*_1_=3.9; *P*=.048) and from EC (*χ^2^*_1_=4.8; *P*=.03) and Northwest China (Fisher exact test: *P*=.008). In addition, posts on offline suicide were more likely than posts on cybersuicide to be coded as *mad* for posts by women (*χ^2^*_1_=5.4; *P*=.02) and from CC (Fisher exact test: *P*=.04) and SC (Fisher exact test: *P*=.03).

### Construction of Classification Models

#### Stigma in General

For exploring linguistic differences in the expression of stigma in general between cybersuicides and offline suicides, to achieve a balanced data set, 600 stigmatizing posts were randomly selected from posts in cybersuicide group (cybersuicide: 600 posts and offline suicide: 588 posts). A total of 6 key features were selected for use in the model construction (Table S2 in Multimedia Appendix 1). The RF model exhibited the best classification performance (precision=0.86, recall=0.85, and *F*-measure=0.85; Table 3).

**Table 3:** Performance of classification models.

Models		Stigma in general	Stupid and shallow	Glorified and normalized
**Naïve Bayes**				
	Precision	0.73	0.72	0.72
	Recall	0.73	0.72	0.72
	*F*-measure	0.73	0.72	0.72
**Support vector machine**				
	Precision	0.83	0.80	0.79
	Recall	0.83	0.80	0.78
	*F*-measure	0.83	0.79	0.78
**Multilayer perceptron neural network**				
	Precision	0.85	0.84	0.78
	Recall	0.85	0.83	0.77
	*F*-measure	0.85	0.83	0.77
**Random forest**				
	Precision	0.86	0.84	0.81
	Recall	0.85	0.84	0.81
	*F*-measure	0.85	0.84	0.81

#### Stupid and Shallow

For exploring linguistic differences in the expression of *stupid and shallow* between cybersuicides and offline suicides, to achieve a balanced data set, a total of 150 *stupid and shallow*–related posts were randomly selected from posts in cybersuicide group (cybersuicide: 150 posts and offline suicide: 129 posts). A total of 4 key features were selected for use in the model construction (Table S2 in Multimedia Appendix 1). The RF model exhibited the best classification performance (precision=0.84, recall=0.84, and *F*-measure=0.84; Table 3).

#### Glorified and Normalized

To explore the linguistic differences in the expression of *glorified and normalized* between cybersuicides and offline suicides (cybersuicide: 148 posts and offline suicide: 195 posts), 28 key features were selected for use in model construction (Table S2 in Multimedia Appendix 1). The RF model exhibited the best classification performance (precision=0.81, recall=0.81, and *F*-measure=0.81; Table 3).

## Discussion

### Principal Findings

To our knowledge, this study provides the first systematic analysis of the differences in stigmatizing attitudes toward cybersuicides and offline suicides. The results of this study have implications for reducing stigma against suicide.

First, it is necessary to confront and reduce stigma against cybersuicide. In this study, a large proportion of cybersuicide-related and offline suicide-related posts were coded as *stigmatizing* (1556/4393, 35.42% and 588/2843, 20.68%, respectively), although most posts about either type of suicide were not overtly stigmatizing. Cybersuicide allows people with suicidal intentions to interact with their audience in the last moments before death. If the audience responds appropriately to people with suicidal intentions, the likelihood of death would be reduced [39-41]. The prevalence of stigma may hamper audience from effectively responding to people with suicidal intentions [25,42-44]. Therefore, cybersuicide deserves to focus on antistigma campaigns.

Second, the public reacts differently to cybersuicides and offline suicides. In terms of attitude types, in this study, cybersuicide was observed to carry more stigma than offline suicide (cybersuicide: 1556/4393, 35.42% and offline suicide: 588/2843, 20.68%; χ^2^_1_=179.8; *P*<.001), implying that the public may react more negatively to cybersuicide than to offline suicide. This is consistent with 2 previous studies that explored audience responses to cybersuicide and offline suicide incidents [24,45]. Furthermore, compared with offline suicide, cybersuicide was more likely to be considered as *stupid and shallow* (*χ^2^*_1_=28.9; *P*<.001) and *false representation* (*χ^2^*_1_=144.4; *P*<.001) and was less likely to be considered as *weak and pathetic* (*χ^2^*_1_=20.4; *P*<.001), *glorified and normalized* (*χ^2^*_1_=177.6; *P*<.001), and *immoral* (*χ^2^*_1_=11.8; *P*=.001). Similar results were also found for different genders and regions. This indicates that antistigma campaigns targeting offline suicide may not be effective in changing stigmatizing attitudes toward cybersuicide, suggesting the need for public awareness campaigns that specifically target cybersuicide. In addition, the most notable and obvious difference between the two types of suicide was found in *false representation* stigma (cybersuicide: 387/1556, 24.87% and offline suicide: 13/588, 2.2%). The misbelief that cybersuicide is not real may influence stigmatizing responses elicited mainly by the value judgment of suicide death itself but not by the perception of suicidal motives, causes, and methods. For example, the glorification and immorality of suicide represent two distinct values: that it is right or wrong to deliberately take one’s own life. However, dismissing cybersuicide as a nonreal condition contradicts the assumption of such 2 stigmas that suicide is a real condition. This might be the reason why cybersuicide was less likely to be considered as *glorified and normalized* and *immoral* than offline suicide. It is also worth noting that cybersuicide was commonly considered as *stupid and shallow* (528/1556, 33.93%) and *false representation* (387/1556, 24.87%), whereas offline suicide was commonly considered as *glorified and normalized* (195/588, 33.2%) and *stupid and shallow* (129/588, 21.9%). According to previous studies, inappropriate audience responses are associated with greater false representation stigma [25], and both suicide ideation and suicide contagion are associated with greater glorification of suicide [46,47]. This indicates that the reduction of stigma against cybersuicide may contribute more in improving audience responses, whereas the reduction of stigma against offline suicide may contribute more in preventing suicide attempts.

Apart from the differences in attitude types, linguistic differences in the expression of stigma between cybersuicides and offline suicides also exist. Such differences existed not only at the level of stigma in general but also at the level of negative stereotypes. In this study, the *F*-measure values of the classification models ranged from 0.81 to 0.85. Compared with other similar studies [48,49], the classification models achieved satisfying accuracy. These results support the conclusion that the way people perceive cybersuicide is very different from the way people perceive offline suicide, implying that cybersuicide may have a different structure from offline suicide [10].

Third, the use of linguistic analysis methods can facilitate the identification of suicide-related stigma in mass media. Mass media is a major contributor to the dissemination of incorrect information, which may reinforce negative stereotypes surrounding mental illness [50-52]. It would be greatly helpful if mass media campaigns were developed to raise public awareness and challenge the stigma against mental illness. However, because of the sheer volume of information in mass media, it is difficult for human coders to identify and analyze stigmatizing information efficiently, suggesting the need for automatic detection of stigma. Linguistic analysis methods can be used to understand language use patterns of stigmatizing expressions and to construct computational models for the automatic detection of stigma [30,33,53]. For example, in this study, stigmatizing expressions of cybersuicide were associated with more frequent use of words related to leisure (eg, chat) and work (eg, dissemination), which may be attributed to the fact that cybersuicide makes suicidal acts highly public and contradicts the public perception of suicide. By contrast, stigmatizing expressions of offline suicide were associated with more frequent use of words related to achievement (eg, hero), which may be attributed to the higher prevalence of suicide glorification (cybersuicide: 148/1556, 9.51% and offline suicide: 195/588, 33.2%). The constructed machine learning models performed well in classifying stigma toward cybersuicides and offline suicides. This indicates that understanding the linguistic differences in stigmatizing expressions between cybersuicides and offline suicides would make automatic detection more precise. Automatic stigma detection would further improve antistigma efforts. The dissemination of internet messages can be helpful in promoting changes in health-related behaviors [54,55]. However, internet campaigns work best when targeted. With the help of automatic detection and machine learning algorithms, it should be easier to efficiently design and deliver target-specific messages to the population and would promote public mental health awareness.

### Limitations

This study has some limitations. First, this study mainly focused on the stigma against livestreamed suicide. Therefore, it is unclear whether these findings are applicable to other types of cybersuicide. Second, social media users are not representative of all people in China. Therefore, the findings may not be applicable to nonusers. Third, the API of Sina Weibo only allowed us to download posts from a certain number of registered users. Therefore, these findings should be further confirmed on a larger scale and in more diverse populations in the future. Fourth, because of the lack of posts obtained from people with suicidal intentions, this study cannot analyze the stigma that people with suicidal intentions put on themselves (ie, self-stigma). Fifth, because of the lack of information about user types (eg, celebrities and general users), this study cannot investigate the differences in attitudes across different types of users and cannot compare attitude responses elicited by the deaths by suicide of different types of users.

### Conclusions

This study used a nonintrusive method to directly and systematically examine the differences in stigmatizing attitudes toward cybersuicides and offline suicides. The results of this study support the conclusion that the way people perceive cybersuicide is very different from the way people perceive offline suicide and offer insight into the reduction of stigma against suicide.

## Appendix

Multimedia Appendix 1 Supplementary Tables S1 and S2.

## Abbreviations

API: application programming interface
CC: Central China
EC: East China
NC: North China
RF: random forest
SC: South China
SWC: Southwest China
