# Supplementary material for: Stigmatizing Attitudes Across Cybersuicides and Offline Suicides: Content Analysis of Sina Weibo
Source: J Med Internet Res. 2022 Apr 8;24(4):e36489. doi: 10.2196/36489 (PMC9034432; doi:10.2196/36489)
Supplement: Multimedia Appendix 1 [file jmir_v24i4e36489_app1.docx]

**Table S1.** Coding framework.

| Categories | | Representative Weibo posts (English translation) |
| --- | --- | --- |
| **Attitudes** | | |
|  | Stigmatizing | - “...People who actually want to kill themselves would not livestream their own suicides to the public...” - “Suicide is a serious crime!” |
|  | Nonstigmatizing | - “God ~~ A livestreamed suicide incident.” - “...Please no more suicides anymore...” |
| **Negative stereotypes** | | |
|  | Weak and pathetic | - “So vulnerable? Livestreaming one’s own suicide on Weibo.” - “...Suicide is the most cowardly act...” |
|  | Self-centered | - “I saw the breaking news regarding the livestreamed suicide incident occurred yesterday...How selfish can a person be...” - “Suicide is a selfish way to kill yourself...” |
|  | Stupid and shallow | - “Livestreaming one’s own suicide on Weibo is the stupidest, stupidest, and stupidest act in the world!” - “People who commit suicides are stupid.” |
|  | False representation | - “...People who actually want to kill themselves would not livestream their own suicides to the public...” - “I think this man committing suicide do not really want to die.” |
|  | Glorified and normalized | - “Livestreaming one’s own suicide on Weibo...So creative.” - “The man committing suicide is brave...” |
|  | Immoral | - “...A livestreamed suicide incident? The God will not grant them repentance...” - “Suicide is a serious crime!” |
|  | Strange | - “...I cannot understand those people livestreaming their own suicides...” - “...I cannot understand why people commit suicide...” |
|  | Embarrassing | - “...People who livestream their own suicides lack a sense of shame...” - “...Suicide is a shame.” |
|  | Vengeful | - “Lady, who do you want to punish by livestreaming your own suicide.” - “...I am strongly opposed to revenge suicide.” |
|  | Mad | - “So mad. Livestreaming one’s own suicide.” - “Suicide is crazy...” |

**Table S2.** Selected key features.

| Comparisons and features | | *t* test (*df*) | *P* value | Cohen *d* |
| --- | --- | --- | --- | --- |
| **Stigma in general (cybersuicide vs offline suicide)** | | | | |
|  | Body | 3.77 (1186) | <.001 | 0.22 |
|  | Work | 12.31 (1186) | <.001 | 0.71 |
|  | Achievement | −4.44 (1186) | <.001 | −0.26 |
|  | Leisure | 21.35 (1186) | <.001 | 1.24 |
|  | Colons | −4.56 (1186) | <.001 | −0.26 |
|  | Other punctuation | −3.82 (1186) | <.001 | −0.22 |
| **Stupid and shallow (cybersuicide vs offline suicide)** | | | | |
|  | Body | 2.41 (277) | .02 | 0.29 |
|  | Sexual | −2.81 (277) | .005 | −0.34 |
|  | Work | 7.62 (277) | <.001 | 0.92 |
|  | Leisure | 9.64 (277) | <.001 | 1.16 |
| **Glorified and normalized (cybersuicide vs offline suicide)** | | | | |
|  | Total function words | −3.71 (341) | <.001 | −0.40 |
|  | Impersonal pronouns | −2.51 (341) | .01 | −0.27 |
|  | Common verbs | −2.48 (341) | .01 | −0.27 |
|  | Prepositions | −5.32 (341) | <.001 | −0.58 |
|  | Conjunctions | −3.41 (341) | .001 | −0.37 |
|  | Signal words for tenses | 2.22 (341) | .03 | 0.24 |
|  | Signal words for present tense | 4.00 (341) | <.001 | 0.43 |
|  | Social processes | −2.15 (341) | .03 | −0.23 |
|  | Humans | −2.88 (341) | .004 | −0.31 |
|  | Positive emotion | −1.99 (341) | .048 | −0.22 |
|  | Negative emotion | −2.26 (341) | .03 | −0.24 |
|  | Cognitive processes | −2.92 (341) | .004 | −0.32 |
|  | Insight | −3.17 (341) | .002 | −0.34 |
|  | Inhibition | −2.04 (341) | .04 | −0.22 |
|  | Inclusive | −2.19 (341) | .03 | −0.24 |
|  | Hear | −2.40 (341) | .02 | −0.26 |
|  | Space | −4.02 (341) | <.001 | −0.44 |
|  | Time | 2.56 (341) | .01 | 0.28 |
|  | Work | 8.88 (341) | <.001 | 0.96 |
|  | Achievement | −3.19 (341) | .002 | −0.35 |
|  | Leisure | 13.69 (341) | <.001 | 1.48 |
|  | Religion | −2.60 (341) | .01 | −0.28 |
|  | Periods | 2.09 (341) | .04 | 0.23 |
|  | Semicolons | −2.27 (341) | .02 | −0.25 |
|  | Quotation marks | −2.59 (341) | .01 | −0.28 |
|  | Apostrophes | 2.39 (341) | .02 | 0.26 |
|  | Word count | −3.01 (341) | .003 | −0.33 |
|  | Words/sentence | −2.12 (341) | .04 | −0.23 |
